# Identification, Design, and Application of Noncoding Cis-Regulatory Elements

**DOI:** 10.3390/biom14080945

**Published:** 2024-08-05

**Authors:** Lingna Xu, Yuwen Liu

**Affiliations:** 1 Shenzhen Branch, Guangdong Laboratory for Lingnan Modern Agriculture, Key Laboratory of Livestock and Poultry Multi-Omics of MARA, Agricultural Genomics Institute at Shenzhen, Chinese Academy of Agricultural Sciences, Shenzhen 518124, China; xulingna@caas.cn; 2 Innovation Group of Pig Genome Design and Breeding, Research Centre for Animal Genome, Agricultural Genomics Institute at Shenzhen, Chinese Academy of Agricultural Sciences, Shenzhen 518124, China; 3 Kunpeng Institute of Modern Agriculture at Foshan, Chinese Academy of Agricultural Sciences, Foshan 528226, China

**Keywords:** CREs, MPRA, predicting CRE activity, de novo designing CREs

## Abstract

Cis-regulatory elements (CREs) play a pivotal role in orchestrating interactions with trans-regulatory factors such as transcription factors, RNA-binding proteins, and noncoding RNAs. These interactions are fundamental to the molecular architecture underpinning complex and diverse biological functions in living organisms, facilitating a myriad of sophisticated and dynamic processes. The rapid advancement in the identification and characterization of these regulatory elements has been marked by initiatives such as the Encyclopedia of DNA Elements (ENCODE) project, which represents a significant milestone in the field. Concurrently, the development of CRE detection technologies, exemplified by massively parallel reporter assays, has progressed at an impressive pace, providing powerful tools for CRE discovery. The exponential growth of multimodal functional genomic data has necessitated the application of advanced analytical methods. Deep learning algorithms, particularly large language models, have emerged as invaluable tools for deconstructing the intricate nucleotide sequences governing CRE function. These advancements facilitate precise predictions of CRE activity and enable the de novo design of CREs. A deeper understanding of CRE operational dynamics is crucial for harnessing their versatile regulatory properties. Such insights are instrumental in refining gene therapy techniques, enhancing the efficacy of selective breeding programs, pushing the boundaries of genetic innovation, and opening new possibilities in microbial synthetic biology.

## 1. Introduction

Historically, genomic research has predominantly concentrated on elucidating the functional implications of protein-coding sequences. Recent advancements in genomic annotation have revealed that over 98% of the human genome comprises noncoding DNA sequences [1]. Genome-wide association studies (GWAS) have further shown that more than 90% of genetic variation lies within these noncoding regions [2]. This previously overlooked portion of the genome is now gaining widespread attention. The National Human Genome Research Institute’s Encyclopedia of DNA Elements (ENCODE) project, initiated in 2003, aims to catalog and characterize the entire repertoire of functional elements within the human genome [3]. This landmark initiative has significantly advanced our understanding of genomic complexity and the regulatory mechanisms that govern gene expression.

The ENCODE Consortium has pioneered robust experimental and bioinformatic methodologies to delineate cis-regulatory elements (CREs) [4,5]. Contemporary high-throughput modalities, including chromatin accessibility assays, histone modification profiling, and three-dimensional chromatin conformation modeling, infer regulatory potential but only indirectly gauge CRE activity through physicochemical indices. To address this, the massively parallel reporter assay (MPRA) was developed, enabling high-throughput quantification of CRE activity using customized plasmid vectors. The MPRA system is comprehensively summarized to enable researchers to flexibly utilize these technologies in conducting related studies.

The systematic identification of regulatory elements has greatly enhanced our understanding of CRE functionalities across various cellular contexts, tissues, species, and temporal states [6,7,8,9,10,11,12,13]. Numerous scholars have developed deep learning models to conduct in-depth CRE analyses [14,15,16,17,18]. These models pose critical questions: Can we predict regulatory elements across species and different spatial and temporal contexts based on sequence homology? What is the syntactic logic of CREs in regulating gene expression? Decoding the key DNA motifs within regulatory elements is foundational for designing these elements, and customizing transcriptional regulation represents a major innovation in biology.

Understanding, identifying, decoding, and designing noncoding regulatory elements are pivotal for advancing research across life sciences, genetic evolution, disease treatment, and animal husbandry and breeding. This review provides an overview of high-throughput technologies for identifying CREs, summarizes studies on predicting and designing regulatory elements using deep learning models, and discusses the potential applications of noncoding regulatory elements in various fields.

## 2. High-Throughput Direct Identification of CRE Activity Using MPRA

Transcription factors (TFs) and their associated cofactors bind to DNA within accessible chromatin domains and exert essential regulatory functions. Advanced genomic methodologies, such as micrococcal nuclease sequencing [19], DNase I hypersensitive site sequencing [20], and assays for transposase-accessible chromatin using sequencing [21], have been pivotal in demarcating these domains that harbor critical CREs, including promoters, enhancers, and silencers. The epigenetic landscape, defined by “core histone modifications”, provides a framework for identifying and decoding functional genomic elements and chromatin organization [22]. Techniques such as chromatin immunoprecipitation-sequencing [23,24,25], cleavage under targets and release using nucleases (CUT&RUN) [26], and cleavage under targets and tagmentation (CUT&Tag) [27,28] use antibodies to recognize histone modifications and specifically capture genomic regulatory element regions. Chromosome conformation capture techniques, such as high-throughput genomic and epigenomic techniques to capture chromatin conformation (Hi-C), offer genome-wide quantification of chromatin contacts [29]. Chromatin interaction analysis with paired-end tag sequencing [30,31] and in situ Hi-C followed by chromatin immunoprecipitation (Hi-ChIP) [32] facilitate the identification of CRE regions. These regions are marked by histone modifications or transcription factor binding. Additionally, 3D genomic approaches provide insights into regulatory element interactions [29] and can elucidate the downstream target genes of such elements [30,31,32].

Despite these advancements, these strategies have inherent limitations and cannot directly localize and quantify CREs with high specificity and precision. Chromatin accessibility assays capture a broad spectrum of regulatory regions. Histone modification capture does not comprehensively encompass all genomic regulatory elements, and three-dimensional interaction maps may include nonfunctional distant contacts as noise. Achieving accurate localization and precise activity measurements of CREs within genomic fragments remains a challenge.

The dual-luciferase reporter system is revered as a gold standard for quantifying CRE activity but is hindered by its low throughput, which is inadequate for the demands of the big data era. Inspired by dual-luciferase plasmid constructs, the MPRA system has been engineered for high-throughput functional interrogation of regulatory elements. MPRA utilizes specific plasmid backbones and regulatory element activity detection strategies based on the functional principles of different regulatory elements. The target genomic fragments are inserted into appropriate screening plasmids, and the CRE activity of a fragment is evaluated by measuring either the transcriptional expression abundance of the tag information paired with the fragment or the transcriptional expression abundance of the fragment itself [33,34,35]. Below, we will detail how researchers use MPRA technology to identify the activity of CREs.

### 2.1. MPRA Promoter

**Figure 1:**
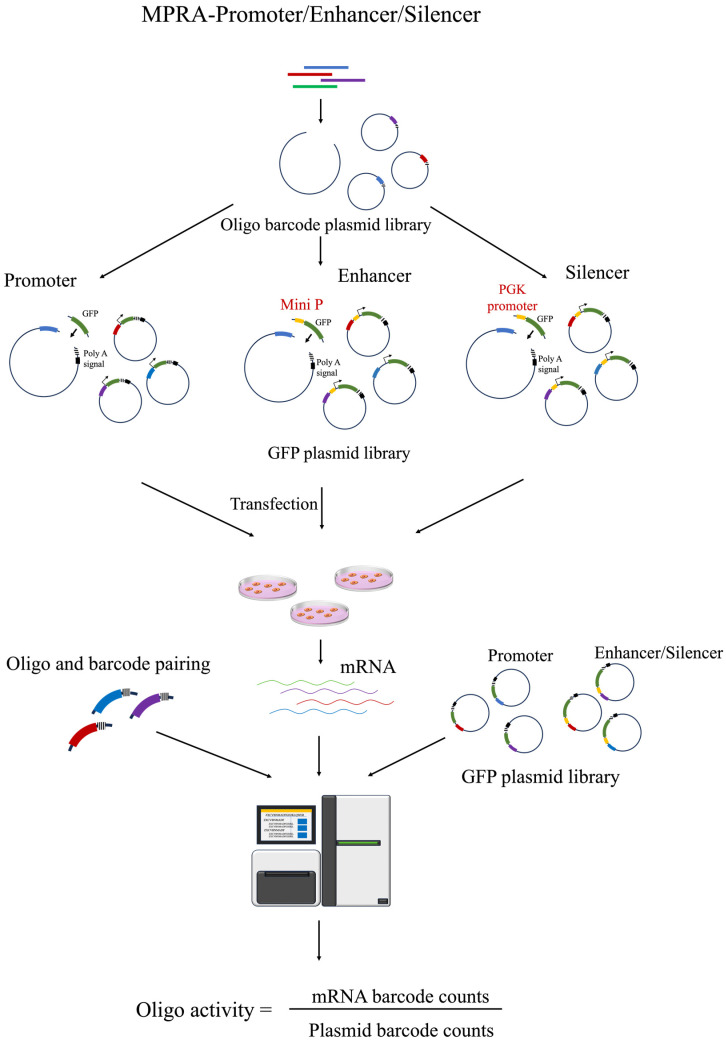
The MPRA system is used for high-throughput identification of promoters, enhancers, and silencers. Initially, a plasmid library pairing test fragments with tags is constructed, followed by the insertion of either GFP alone or a promoter followed by GFP. This GFP plasmid library is then transfected into cells, after which cellular RNA is collected to create an RNA-seq library. The activity of the test fragments is determined by comparing the ratio of tag counts between the GFP plasmid library and the RNA-seq library using the pairing information of the test fragments and tags. The differentiation among them is that only GFP is inserted for promoter activity testing, miniP and GFP for enhancers, and a strong promoter such as PGK plus GFP for silencers.

Promoter activity test fragments were systematically paired with unique barcodes and integrated into a plasmid backbone, incorporating a fluorescent reporter gene, such as green fluorescent protein (*GFP*), to facilitate high-throughput screening (Figure 1). We constructed a plasmid library dedicated to promoter identification, termed the input library, which was subsequently sequenced. The library was transfected into specific cell lines, and RNA was extracted to construct an RNA-seq library for sequencing, which is referred to as the output library. The promoter activity of each test fragment was quantitatively assessed by analyzing the normalized output-to-input library ratio of the corresponding barcodes.

Ryan et al. leveraged the MPRA system to identify variants that modulate gene expression by influencing promoter activity, thereby contributing to a genetic predisposition to ankylosing spondylitis [36]. In a related study, van Arensbergen et al. employed this approach on a genome-wide scale in K562 cells to map active gene promoters [37]. Furthermore, Zhao et al. advanced this technique by integrating single-cell technology with MPRA and developed single-cell MPRA to pinpoint promoter activity-affecting variants across distinct cell subtypes within the mouse retina [8].

### 2.2. MPRA Enhancer

**Figure 2:**
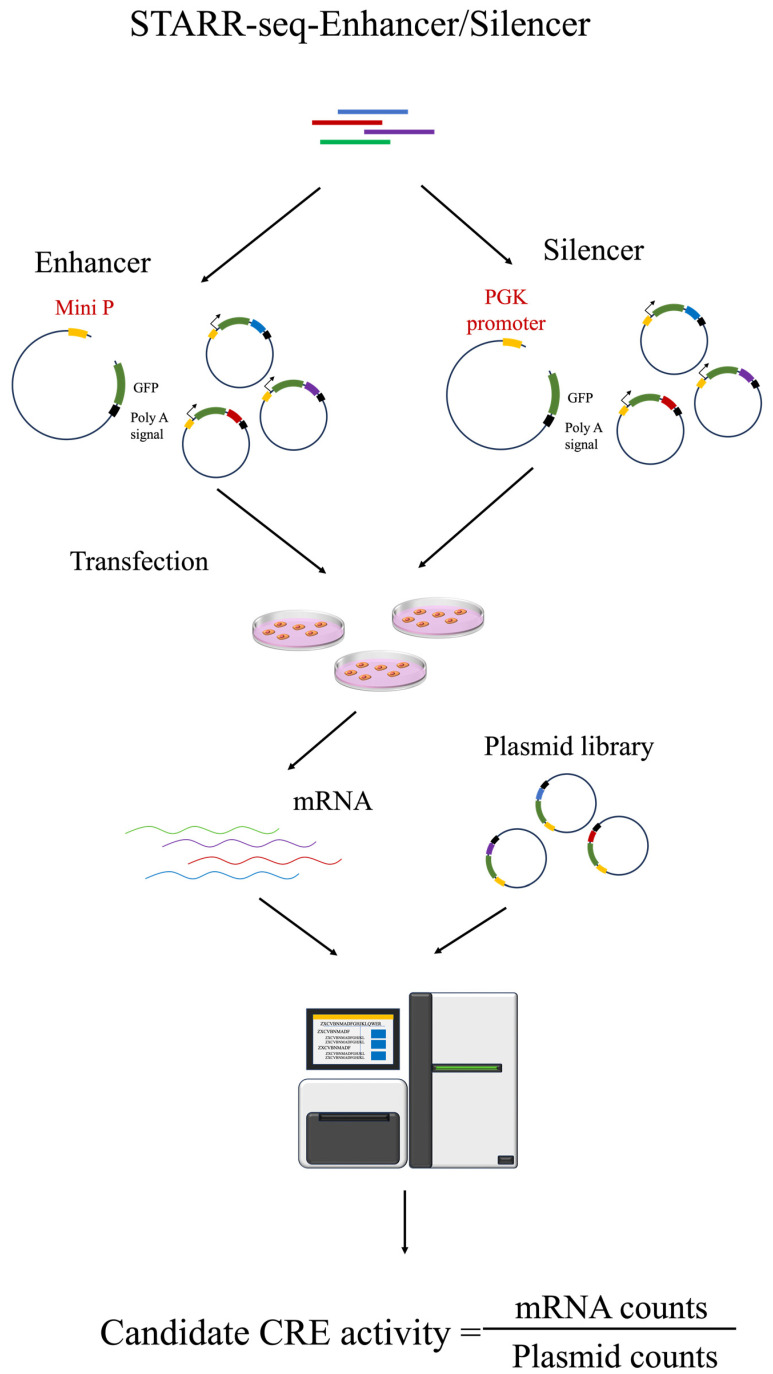
The STARR-seq system is employed for high-throughput identification of enhancers and silencers. Test fragments are inserted between the GFP and poly A signal within a plasmid backbone, and the plasmid library is sequenced and transfected into cells. An RNA-seq library is then constructed, and the activity of enhancers or silencers is quantified by calculating the abundance ratio of the transcript inserts relative to the plasmid library. The main distinction between the identification of these elements lies in the type of the promoter used; enhancers use a weak promoter such as miniP or ORI, while silencers use a strong promoter like PGK.

Enhancer library construction parallels that for promoters but involves the integration of a minimal promoter (miniP) and a *GFP* reporter gene between the test fragment and barcode (Figure 1). This setup facilitates a high-throughput screening system for enhancer activity [33]. Unlike promoters, enhancers can exert regulatory influence without directional or proximal constraints. To explore this, Stark et al. developed self-transcribing active regulatory region sequencing (STARR-seq), a derivative of MPRA, in which enhancer fragments are cloned downstream of a minimal promoter and upstream of a polyadenylation signal within a plasmid (Figure 2). In cellular environments, an active enhancer sequence interacts with the promoter to drive the concurrent expression of the reporter gene and the enhancer sequence itself, which could be directly measured through RNA-seq. With the capability to screen DNA fragments at a whole-genome scale, this method has been first applied to map enhancer activity throughout the *Drosophila* genome, providing substantial insights into the enhancer function [17,38,39,40].

STARR-seq offers several advantages over traditional MPRA, including its applicability to native genomic fragments such as randomly sheared whole-genome fragments, chromatin immunoprecipitated DNA, or enriched open chromatin areas. These fragments can be efficiently integrated into a STARR-seq vector, simplifying library construction and reducing costs while increasing throughput (Figure 2). Liu et al. successfully applied STARR-seq to delineate and quantify enhancer activity across the mammalian genome, achieving a system throughput of 158.6 million sequences [40]. Subsequent innovations have extended the utility of STARR-seq, leading to the development of several variants such as Capture-STARR-seq [41,42], ATAC-STARR-seq [43,44], ChIP-STARR-seq [45], population-scale STARR-seq assays [46], DNA methylation-STARR-seq [47], biallelic targeted STARR-seq [48], and massive active enhancers by sequencing [49]. Each of these technologies addresses specific challenges and expands the scope of their potential applications.

The advent of STARR-seq using adeno-associated viruses [50,51] has facilitated high-throughput identification of enhancer activities in vivo, although the efficiency of this method is currently limited by adeno-associated-viruses (AAV) transduction rates. Enhancements in AAV capsid technology, particularly those that optimize tissue-specific transduction [52], are expected to significantly enhance the throughput and applicability of this approach. Moreover, the integration of nanotechnology for in vivo delivery promises safer and more efficient applications.

Finally, the application of STARR-seq in single-cell formats provides unique challenges, notably the dispersion of limited plasmid transcripts across diverse cell subtypes, which complicates the capture of limited transcriptomic data. Mangan et al. addressed this issue by employing semi-nested polymerase chain reaction (PCR) to selectively amplify transcript information from various cell subtypes, thereby facilitating the detection of enhancer activity in heterogeneous cell populations [53].

### 2.3. MPRA Silencer

Silencers, akin to enhancers, exert regulatory functions irrespective of spatial and positional constraints and can be characterized through high-throughput methodologies such as STARR-seq. However, a critical difference lies in promoter selection: for enhancer identification, weak promoters or elements with inherently low promoter activity are preferred to enhance the sensitivity of detection [54,55]. Conversely, robust promoters, such as phosphoglycerate kinase (*PGK*) or super core promoter 1 (*SCP1*), are indispensable for the effective identification of silencers (Figure 1 and Figure 2). Jayavelu et al. employed a STARR-seq platform utilizing the *SCP1* promoter to identify and catalog approximately 7500 potential silencing fragments across human and mouse genomes within K562 cell lines [56]. Further exploring this paradigm, Saadat Hussain used three distinct STARR-seq vectors incorporating *SCP1*, *PGK*, and the lymphoid-specific recombination-activating gene 2 (*Rag2*) gene promoter to evaluate silencer activity within mouse DNase I hypersensitive sites regions in T cell lines, revealing that more potent promoters, such as *PGK* and *Rag2*, are superior in detecting a greater number of silencers [57]. Hansen et al. applied ATAC-STARR-seq to a female B-cell lymphoblastoid cell line (GM12878) to concurrently assess the activity of both enhancers and silencers [44], a method that leverages the capacity of this system to detect dual regulatory elements within a singular experimental setup. Although using the ATAC-STARR-seq system to simultaneously identify enhancers and silencers is highly innovative, the promoter activity of the ORI element in the ORI-STARR-seq backbone is quite weak, which may result in some false positives when identifying silencers [58].

### 2.4. MPRA Insulator

**Figure 3:**
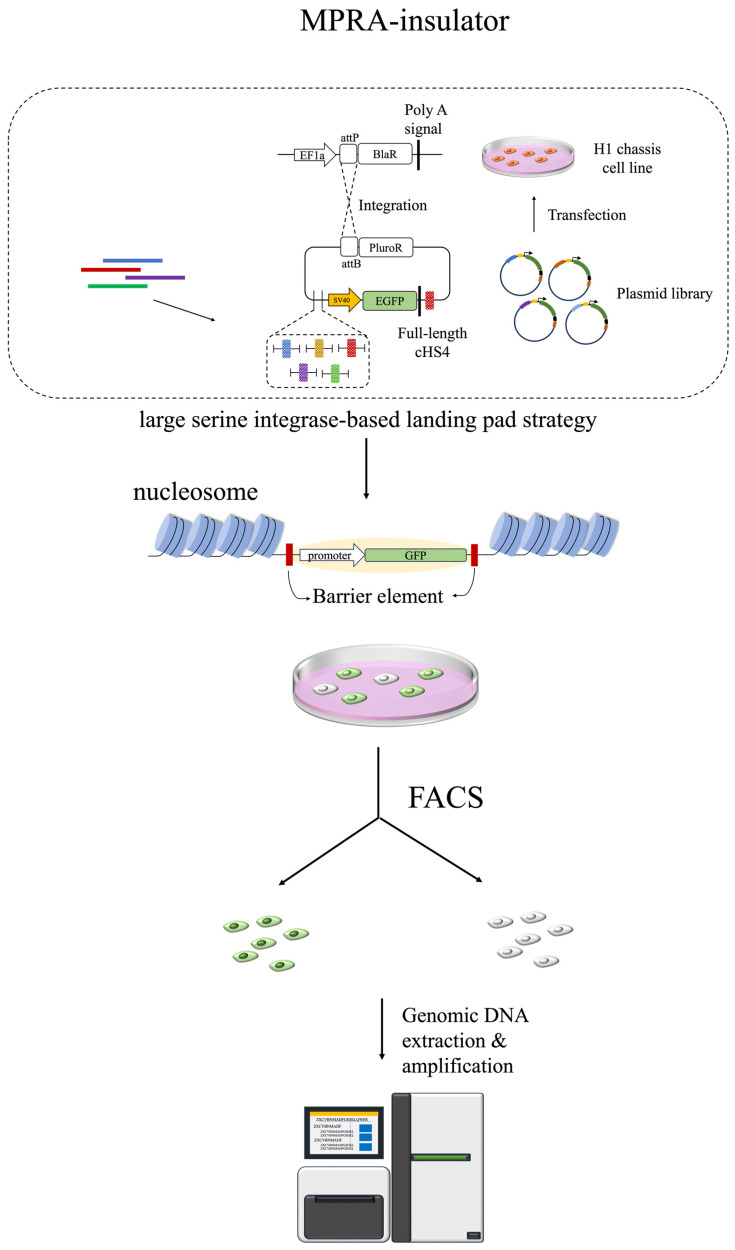
High-throughput identification of insulators using the MPRA system. This system assesses the regulatory impact of isolated heterochromatin on gene function. Developed by Zhang et al., it employs serine integrase to integrate test sequences near heterochromatin and analyzes the shielding effect of test fragments through the expression intensity of target genes such as *GFP* [9].

Insulators play pivotal roles in genomic architecture through two principal mechanisms: first, by functioning as enhancer blockers, they prevent enhancers from modulating adjacent promoters; second, they serve as barriers that insulate genes from the transcriptional interference of heterochromatin [59,60,61]. To further explore and characterize these elements, Zhang et al. developed a site-specific heterochromatin insertion of elements in the lamina-associated domain platform. This approach leverages a serine integrase-based strategy for the high-throughput identification of insulators capable of mitigating the influence of heterochromatin on gene transcription, offering new insights into the spatial regulation of gene expression [9] (Figure 3). Hong et al. developed a massively parallel integrated regulatory elements (MPIRE) framework and used it to measure the insulator effects of three insulators—CTCF binding sites (A2 and cHS4) and B box motifs (ALOXE3)—as well as their mutants at thousands of locations in the genome. The study found that while all three insulators could block enhancers in the genome, only ALOXE3 could act as a heterochromatin barrier [62].

### 2.5. MPRA-5′

**Figure 4:**
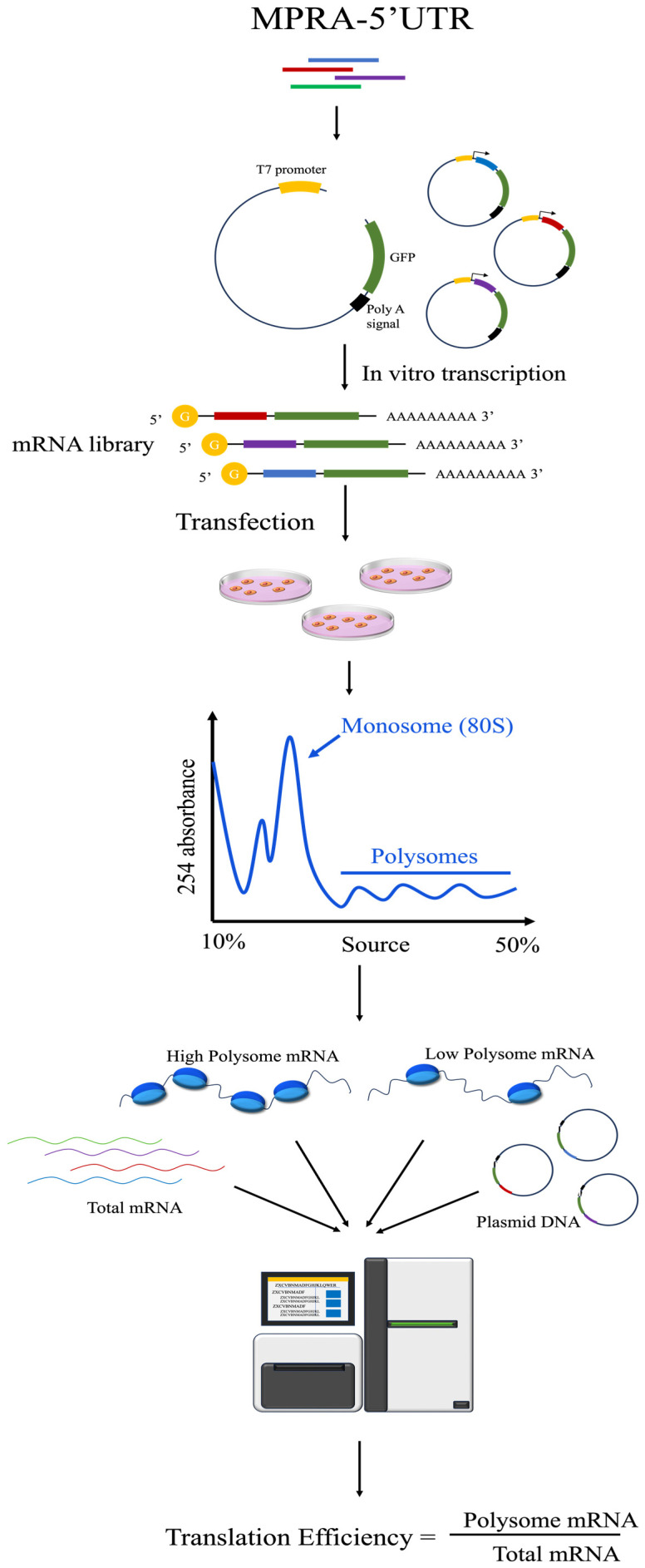
High-throughput identification of 5′ UTRs using the MPRA system. This strategy combines MPRA with in vitro transcription and ribosome component analysis to identify the impact of 5′ UTRs on the protein translation process.

The 5′ untranslated region (5′ UTR) plays a crucial role in the regulation of gene transcription and translation. To investigate this, researchers typically integrate target sequences downstream of a promoter and upstream of a reporter gene within an MPRA framework. This setup facilitates the concomitant application of in vitro transcription, ribosome profiling, and RNA sequencing to quantitatively assess the influence of 5′ UTRs on gene transcription on a broad experimental scale [16,63,64,65,66,67] (Figure 4). Considering the variable length of 5′ UTRs, which ranges from 18 to >3000 bases, and the profound impact of both UTR length and sequence context on gene expression, Yiting Lim and associates have developed a pooled full-length UTR multiplex assay on gene expression. This innovative approach uses single-molecule real-time sequencing to capture paired full-length 5′ UTRs and downstream barcodes. Subsequent barcode sequencing of DNA, total mRNA, and ribosome-associated mRNA enables a detailed analysis of the regulatory effects exerted by 5′ UTRs on both transcription and translation processes [68].

### 2.6. MPRA-3′ UTR

**Figure 5:**
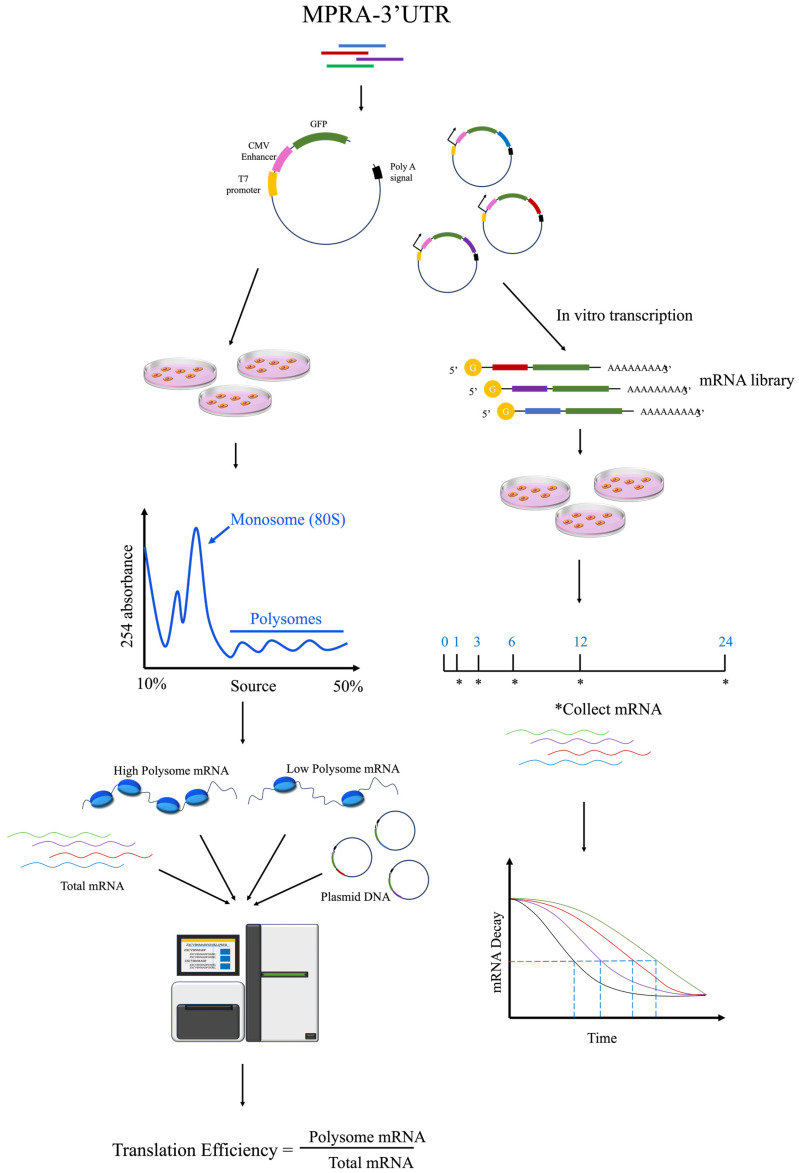
High-throughput identification of 3′ UTRs using the MPRA system. The MPRA system uses MPRA and ribosome component analysis to study the impact of 3′ UTRs on protein translation, employing in vitro transcription to collect mRNA at different time points to study the impact of 3′ UTRs on mRNA stability. The asterisks indicate the time points at which mRNA was collected.

Contemporary MPRA systems, widely used for the high-throughput characterization of 3′ untranslated regions (3′ UTRs), adopt a configuration akin to the STARR-seq system. These assays strategically position test fragments downstream of a promoter and reporter gene and upstream of a poly A termination signal. Diverging from the STARR-seq framework, which employs relatively weak promoters for enhancer detection, MPRA systems for 3′ UTRs incorporate robust promoters to enhance transcriptional output. Oikonomou et al. implemented a dual-fluorescence system harnessing the strengths of *GFP* and *mCherry*, both driven by potent promoters. The test fragments were integrated downstream of the *mCherry* reporter gene, facilitating the identification of regulatory elements within the 3′ UTR via fluorescence-activated cell sorting [69]. Further advancements were made by Dustin Griesemer, who developed the MPRAu system using the strong PGK promoter to drive transcriptional machinery. This system was engineered to insert test fragments downstream of the *GFP* reporter gene, enabling evaluation of the functional impacts of 12,173 3′ UTR variants linked to human diseases and evolutionary traits across six distinct cell lines [70]. These plasmid-based configurations allow for a systematic analysis of the regulatory effects mediated by 3′ UTRs, offering a streamlined approach to genomic research. However, further investigations are necessary to elucidate the intricate dynamics of post-transcriptional mRNA translation and stability. The development of the Fast-UTR system, predicated on a bidirectional tetracycline-regulated viral reporter gene, has facilitated the quantification of the effects of 3′ UTR sequences on both mRNA and protein synthesis [71,72,73]. MPRA system is combined with in vitro transcription for high-throughput analysis of 3′UTR regulation of mRNA stability [71,74,75] (Figure 5).

### 2.7. MPRA Technology Is an Effective Strategy for Fine-Mapping Causal Variants of Complex Traits

Comprehensive GWASs have been undertaken to elucidate the influence of genetic variants on complex traits. These studies have often revealed that loci associated with quantitative traits display extensive linkage disequilibrium (LD), encompassing numerous DNA variants that affect phenotypes across genome-wide significance regions. Nevertheless, traditional GWAS methodologies often fail to identify precise causal variants responsible for trait variations. To address this limitation, experimental and computational strategies have been employed to facilitate the identification of causal variants at specific loci. Advanced fine-mapping techniques that leverage association analysis and LD models have been developed to enhance the prediction of potential causal variants [76,77,78,79]. Despite these advancements, heterogeneity in computational predictions necessitates further experimental validation to confirm true causal variants. Innovative methods, such as allele-specific chromatin accessibility, have been implemented to assess how genetic variations influence chromatin accessibility within pertinent cell types, thereby affecting gene expression and phenotypes [80,81]. Similarly, single nucleotide polymorphism (SNP)-ChIP techniques have been used to identify SNPs that affect CREs using genetically diverse donor materials [82]. However, these techniques often reveal that multiple SNPs regulate a single CRE, complicating the isolation and study of individual variant effects.

The adoption of MPRA has significantly advanced the field by enabling high-throughput validation of thousands of genetic variants for their regulatory activity, thereby fine-mapping causal variants that influence diseases or complex traits [83,84,85]. Recent research has increasingly focused on the identification of functional regulatory variants within GWAS loci, using both historical data and novel investigative techniques [11,36,86,87,88,89,90,91,92,93,94,95]. Ryan et al., in a pivotal 2016 study, used a promoter MPRA system to screen approximately 30,000 expression quantitative trait locus (eQTL) variants in B lymphoblastoid cell lines and identified 842 variants with allele-specific expression differences [36]. Enhanced MPRA systems have been widely applied to investigate the regulatory roles of genetic variation across a diverse array of diseases and conditions, including schizophrenia [11,92,95], Alzheimer’s disease [95], obesity [86], melanoma [89], multiple myeloma [88], skin pigmentation disorders [93], Lassa fever [94], and eosinophilic esophagitis [96]. Furthermore, Duan et al. leveraged the STARR-seq system to assess enhancer activity for nearly 6000 insulin resistance-related GWAS variants in HepG2 liver cancer cells, preadipocytes, and A673 rhabdomyosarcoma cells, demonstrating the practical applications of these techniques in functional genomics [91]. Abell et al. employed the MPRA system to investigate the regulatory activities of eQTLs and GWAS loci under both single and linked genetic conditions and uncovered the predominant additive effects in most haplotype combinations [87]. These studies underscore the critical role of innovative genomic technologies in advancing our understanding of the genetic determinants of complex biological traits.

### 2.8. Limitations of MPRA

Although the MPRA system is a highly effective tool for high-throughput identification of CREs and regulatory variants, it has several limitations that affect its utility in functional genomics. One primary constraint is the inability to ascertain which genes are affected by CREs and regulatory SNPs. This limitation necessitates the integration of MPRA findings with other functional genomic datasets, such as eQTLs and Hi-C, to elucidate the genomic targets of these regulatory elements [97]. Furthermore, the MPRA approach predominantly employs exogenous plasmid constructs to assess regulatory activity and fails to accurately replicate the native chromatin context of the genome. Research has indicated that a fraction of the regulatory elements identified via MPRA are situated within heterochromatin regions, potentially rendering them nonfunctional in their natural genomic environments [40]. Consequently, the application of MPRA to identifying functional CREs and variants requires thorough validation by incorporating additional epigenomic factors, such as chromatin accessibility and histone modifications. Integrative analyses are critical to accurately assessing the functional relevance of regulatory elements in the complex architecture of the genome.

The MPRA system typically identifies regulatory element activity by calculating the ratio between the RNA-seq coverage and the genome sequencing coverage of a fragment to be tested. Processing duplicate reads is a dilemma in RNA-seq experiments, particularly in the context of MPRA. Retaining duplicates risks being affected by PCR amplification efficiency, while discarding duplicates risks underestimating the abundance of highly expressed sequences. Our experience indicates that for whole-genome screening MPRA, where transcript abundance is lower for each potential CRE, collapsing duplicated reads is preferred. Conversely, for target MPRA focusing on tens of thousands or fewer fragments, retaining duplicated reads is favored.

Additionally, a more effective, albeit technically challenging, approach is to add unique molecular identifiers (UMIs) to the library before the PCR step during library construction [17,91,98]—for example, adding UMIs to the plasmid library and reverse transcription products before PCR of sequencing adapter attachment. During data analysis, deduplication based on UMIs can effectively distinguish between the inherent genomic and transcriptomic information of the library and the duplicates from library construction and sequencing PCR, accurately identifying the activity of regulatory elements. However, for highly complex MPRA libraries, the difficulty of adding UMIs increases significantly, and a large amount of sequencing data is required to comprehensively detect all UMI information.

## 3. Using Deep Learning for Predicting CRE Activity and De Novo Design

Since the deep learning renaissance in 2012, deep learning has propelled a revolution across various domains, championing data-centric methodologies. Deep learning uses deep neural networks, which are sophisticated constructs comprising numerous layers of artificial neurons. The crucial advantage of deep learning is embedding nonlinear feature computations within the architecture of the model. This facilitates the identification of intricate patterns hidden in expansive datasets [99,100]. High-throughput functional genomics technologies have yielded a substantial high-quality dataset of CREs, rendering the application of deep learning models for CRE analysis feasible and highly effective.

### 3.1. Deep Learning Models Are Used to Predict the Activity of CREs and mRNA Expression Levels

Recent breakthroughs have catalyzed the development of deep learning models and considerably enhanced genomic analytics. These models predict the DNA and RNA protein-binding affinities and activity of CREs. The DeepBind model [101] is used for the precise prediction of protein-DNA/RNA interactions by decoding nucleotide sequence patterns. Employing convolutional neural network architecture to predict expression levels in promoter or terminator regions reveals that the 3′ UTR can subtly adjust mRNA expression, whereas the 5′ UTR has a substantial impact on altering mRNA expression levels [102]. The Xpresso model uses genomic sequences to estimate mRNA expression levels, enabling the quantification of the influence exerted by enhancers, heterochromatic domains, and microRNAs [103]. In parallel, the model for the 5′ UTR language model accurately estimates transcript abundance, revealing insights into gene regulation and mRNA synthesis [15]. These tools are particularly adept at identifying known binding sites and discovering novel interactions, thereby elucidating the intricate regulatory mechanisms of gene expression. In addition, owing to their high predictive accuracy, deep learning models offer crucial insights into the complexities of genetic disorders. Tools such as DeepSEA [104] and Basset [105] predict the impact of genetic variants, such as SNPs and insertion-deletions (InDels), on the activity of enhancers and other CREs. Furthermore, by integrating them with human GWAS datasets, they identified disease-associated causal SNPs [106,107], underscoring their value in unraveling the genetic underpinnings of diseases.

### 3.2. Deep Learning Models Decode Sequence Features of CREs and Accurately Predict Activity

Deep learning architectures, which are intricate and nonlinear, are misunderstood as opaque “black boxes”. However, unraveling the DNA sequence characteristics that these models learn—how they synthesize these features to precisely predict CRE activity—broadens our understanding of the CRE regulatory syntax. Progress in model interpretability has highlighted the pivotal role of DNA motifs in the function of CREs, along with their spatial orientation, distribution, and interactions, thus unraveling regulatory grammar. For example, the DeepSTARR model delineates enhancer-associated TF motifs and complex syntax rules, highlighting functional variability within identical TF motifs based on flanking sequences and motif spacing [17]. The FactorNet model uses deep neural networks to predict cell type-specific binding to TFs [108]. DeepSEA [104], Basset [105], and Enformer [109] have systematically decoded the genomic syntax for regulatory regions and histone modifications. Cross-species models further demonstrate the conservation of TF-binding preferences, thereby enriching our understanding of fundamental aspects of gene regulation. The DeepMEL model was developed to decode the syntactic structures of enhancers across various species, thereby enabling the prediction of enhancers in additional cell types and species [110]. Sethi et al. devised a STARR-seq model to decipher the syntactic information of enhancers in fruit flies, which they subsequently applied to predict enhancers in mammalian species [111]. The DeepArk model uses epigenomic data from four model organisms to extensively examine the influence of DNA sequences on CRE activity. This model predicts the regulatory effects of genomic variations [112].

### 3.3. De Novo CREs Design Using Learning Models

Given the ability of deep learning models to predict CRE activity directly from DNA sequences in silico, they substantially enhance the efficiency of traditional mutagenesis-based designs. For instance, DeepSEED is exemplary for refining the context of TF-binding sites, which is a key aspect of efficient promoter engineering [113]. Meanwhile, the DeepSTARR model by the Stark lab and cell type-specific enhancers by the Aerts group exemplify the transformative power of deep learning in enhancer design [14,17]. Furthermore, optimizing UTRs using deep learning techniques is critical for boosting protein expression and improving mRNA therapy and vaccine efficacy [18]. Pioneering work by Zeng et al. produced NCA-7d as the 5′ UTR and S27a plus a functional motif R3U as the 3′ UTR (named as NASAR) combination of UTRs, offering an efficiency leap over traditional UTRs [114]. Tang and colleagues’ Smart5 UTR model, integrating N1-methyl-pseudouridine into 5′ UTRs, has significantly advanced mRNA vaccine effectiveness against challenging viral variants [115]. The de novo design of regulatory elements allows for precise modulation of gene expression across varying levels of activity, which is crucial for the development of targeted gene therapies.

### 3.4. Challenges of Integrating Deep Learning with MPRA

Integrating deep learning with MPRA technology can substantially enhance the precision and robustness of cis-regulatory elements and gene regulation predictions. MPRA generates extensive datasets that are invaluable for training deep learning models, particularly for predicting TF binding, enhancer activity, and gene expression levels [64,67,74,116,117,118,119,120]. However, such integration presents several challenges. First, to meet the requirements of deep learning models, MPRA experiments usually necessitate larger library sizes. For instance, when the number of fragments to be tested reaches 100,000 or 1 million, and the library complexity after pairing with barcodes reaches 10 million or 100 million, the experiment becomes highly challenging [117,119]. Second, the interpretability of deep learning models developed for different MPRA datasets is still in the exploratory stage [105,121,122]. The high dimensionality of data increases the risk of overfitting, necessitating the use of regularization techniques, cross-validation, and data augmentation strategies to mitigate this issue [17,123]. Additionally, the diversity of the training data may be limited, necessitating broader MPRA experiments to create more comprehensive datasets. Future research should prioritize the integration of multi-omics data, the development of transfer learning approaches, and the advancement of methods for model interpretability. Innovations in MPRA technology, such as optimized library design and advanced readout methodologies, will further enhance data quality and quantity [98,124,125]. Addressing these challenges and leveraging the synergistic strengths of MPRA and deep learning will lead to significant advancements in elucidating complex genomic regulatory landscapes and their effects on gene expression.

## 4. Outlook: Broad Applications of CREs across Various Fields

**Figure 6:**
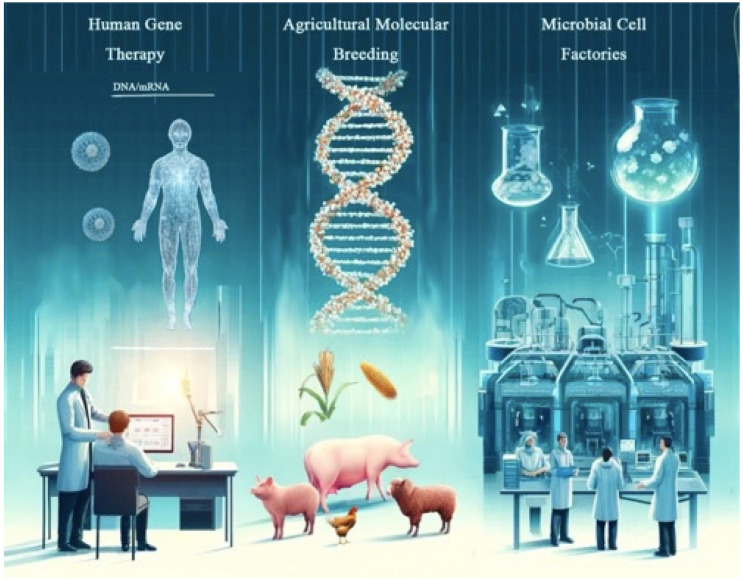
Applications of CRE research in human gene therapy, agricultural molecular breeding, and microbial cell factories. The left side of the diagram shows how humans integrate the research findings of CREs with human gene therapy technologies to develop customized treatment plans for human diseases. The middle part of the diagram illustrates the use of identified functional sites that affect economically important traits for breeding superior varieties or the use of functional elements that influence traits for gene editing to cultivate high-quality, high-yield varieties. The right side of the diagram displays the continuous optimization and upgrading of the production processes and yields of microbial cell factories through the optimization and modification of CREs.

As research into CREs advances, their mechanisms of action are gradually being elucidated. Benefiting from the rapid progress in this field, fine-tuning CRE activities to control gene transcription and translation havehas broad applications across numerous areas (Figure 6).

### 4.1. Optimizing CREs Is One of the Key Future Research Directions for Human Gene Therapies

Human gene therapy encompasses two primary strategies: (1) the introduction of DNA, mRNA, or proteins into the body via vectors such as AAV or lipid nanoparticles to replace defective genes and sustain essential biological functions and (2) employing the CRISPR/Cas9 system for precise gene editing to rectify pathogenic mutations or using CRISPR/dCas9 to modulate the expression of disease-related genes. Enhancing each component of these gene therapy strategies can substantially improve therapeutic efficacy.

For DNA therapy, optimizing regulatory elements, such as promoters and enhancers, can significantly boost the transcription and expression levels of introduced genes. In mRNA therapy, refining UTR sequences can increase protein translation efficiency, thereby enhancing the therapeutic potential of mRNA-based treatments [18,114,115].

When considering delivery systems such as AAV, the design of tissue- or cell subtype-specific promoters or enhancers can facilitate targeted gene expression, thereby minimizing off-target effects on non-diseased tissues or cells [126,127,128]. Similarly, for gene-editing systems, the optimization of regulatory elements can lead to greater editing precision and efficiency [129,130].

### 4.2. Optimizing CREs Is Critical for the Development of Agricultural Crops and Livestock Breeds with Superior Quality and Yield

In agriculture, epigenomic research on livestock [6,131,132,133,134,135] and crops [136,137] has integrated genomic and transcriptomic data to annotate complex trait GWAS loci, thereby providing essential genetic markers for breeding programs. Breeding efforts are often constrained by trade-offs between complex traits such as meat production versus intramuscular fat in pigs or milk yield versus protein content in dairy cows. A breakthrough study by Song et al. used CRISPR-Cas9 to induce a 54-bp deletion in the rice *IPA1* gene promoter, which simultaneously increased tiller number and panicle size, thus overcoming the inherent trade-offs [138]. This suggests the potential for marker-assisted selection and the use of gene and CRE editing to improve crop and livestock varieties for enhanced yield and quality.

### 4.3. In-Depth Research on CREs Lays a Solid Theoretical Groundwork for Customizing Microbial Cell Factories in the Future

In the field of synthetic biology, microbial biomanufacturing has garnered increasing interest in the production of diverse products [139]. Microbial cell factories use microbes as platforms to produce fuels and chemicals, and there is an increasing focus on optimizing CREs within bacterial plasmid backbones to enhance gene expression [140] and increase the expression of yeast proteins [141]. Future research could enable the custom design of CREs that are responsive to temperature, light, and pH, tailoring microbial production to diverse environmental conditions and achieving precise control over industrial outputs.

### 4.4. Conclusions and Overlook

The study of noncoding functional CREs is intensely active, and a plethora of experimental and analytical methods are continuously being developed. However, the interactions between CREs and trans-regulatory factors, as well as interactions among different CREs, are highly complex. Current experimental strategies for identifying CREs and their interactions are still not fully developed. This necessitates the ongoing advancement of new methods or the integration of multi-omics technologies to further elucidate the molecular mechanisms and sequence regulatory grammars of CREs. Deep learning models for predicting and designing regulatory elements require continuous development and adjustment of algorithms, as well as more high-quality experimental data to expand the training sets and improve prediction accuracy. As research into CREs progresses, we anticipate that the functional annotation and regulatory mechanisms of CREs will become clearer. With continuing technological advancements across various disciplines, noncoding CREs are poised for significant breakthroughs in an increasing number of areas.
